# Sestrin2 protects against cholestatic liver injury by inhibiting endoplasmic reticulum stress and NLRP3 inflammasome-mediated pyroptosis

**DOI:** 10.1038/s12276-022-00737-9

**Published:** 2022-03-08

**Authors:** Daewon Han, Haeil Kim, Soojin Kim, Qui Anh Le, Seung Yun Han, Jeongyun Bae, Hye Won Shin, Hyun-Goo Kang, Kyung Ho Han, Jongdae Shin, Hwan-Woo Park

**Affiliations:** 1 Department of Cell Biology, Konyang University College of Medicine, Daejeon, 35365 Republic of Korea; 2 Department of Anatomy, Konyang University College of Medicine, Daejeon, 35365 Republic of Korea; 3 Department of Biological Sciences and Biotechnology, Hannam University, Daejeon, 34054 Republic of Korea; 4 Myunggok Medical Research Institute, Konyang University College of Medicine, Daejeon, 35365 Republic of Korea; 5 Present Address: Department of Anatomy, Catholic Neuroscience Institute, College of Medicine, The Catholic University of Korea, Seoul, 06591 Republic of Korea; 6 Present Address: Cardiovascular Research Center and Department of Cardiovascular Sciences, Temple University School of Medicine, Philadelphia, PA 19140 USA

**Keywords:** Mechanisms of disease, Apoptosis, Stress signalling

## Abstract

Chronic exposure to bile acid in the liver due to impaired bile flow induces cholestatic liver disease, resulting in hepatotoxicity and liver fibrosis. Sestrin2, a highly conserved, stress-inducible protein, has been implicated in cellular responses to multiple stress conditions and the maintenance of cellular homeostasis. However, its role in cholestatic liver injury is not fully understood. In this study, we investigated the role of hepatic Sestrin2 in cholestatic liver injury and its underlying mechanisms using in vivo and in vitro approaches. Hepatic Sestrin2 expression was upregulated by activating transcription factor 4 (ATF4) and CCAAT/enhancer-binding protein-β (C/EBP-β) after treatment with bile acids and correlated with endoplasmic reticulum (ER) stress responses. Bile-duct ligation (BDL)-induced hepatocellular apoptosis and liver fibrosis were exacerbated in Sestrin2-knockout (*Sesn2*^−/−^) mice. Moreover, Sestrin2 deficiency enhanced cholestasis-induced hepatic ER stress, whereas Sestrin2 overexpression ameliorated bile acid-induced ER stress. Notably, the mammalian target of rapamycin (mTOR) inhibitor rapamycin and the AMP-activated protein kinase (AMPK) activator AICAR reversed bile acid-induced ER stress in Sestrin2-deficient cells. Furthermore, Sestrin2 deficiency promoted cholestasis-induced hepatic pyroptosis by activating NLRP3 inflammasomes. Thus, our study provides evidence for the biological significance of Sestrin2 and its relationship with cholestatic liver injury, suggesting the potential role of Sestrin2 in regulating ER stress and inflammasome activation during cholestatic liver injury.

## Liver disease: Minimizing the stress from bile blockage

A protein that manages the response to cellular stress can help prevent disruptions in bile flow from progressing to liver fibrosis or failure. Disrupted flow leads to the accumulation of bile acids, which triggers a state known as endoplasmic reticulum (ER) stress, fueling inflammation and eventual cell death. Researchers led by Hwan-Woo Park and Jongdae Shin at Konyang University, Daejon, South Korea, have demonstrated that the Sestrin2 protein plays a prominent role in managing this ER stress response to cytotoxic bile acids in cultured liver cells. They subsequently used a Sestrin2-deficient mouse model to demonstrate that the absence of this protein contributes to heightened ER stress and greatly increased liver damage following impaired bile flow. These results suggest that Sestrin2 modulators could offer effective treatments for liver disorders associated with bile flow obstruction.

## Introduction

Bile acids are amphipathic molecules synthesized in hepatocytes as the major products of cholesterol catabolism and act as biological detergents that promote the absorption, transport, and distribution of lipids and fat-soluble vitamins^1^. Bile acids are also required to modulate metabolic pathways, including glucose metabolism, lipid metabolism, and energy expenditure^2^. Impaired bile flow, due to blockage of the biliary tract or genetic defects, causes the accumulation of bile acids in the liver and systemic circulation and results in cholestatic liver diseases such as cholestasis, which leads to progressive liver fibrosis and ultimately liver failure^3–5^. Despite progress in understanding the physiological events, the cellular mechanisms by which bile acids induce liver injury remain elusive.

Endoplasmic reticulum (ER) stress is implicated in the etiology of several liver diseases, including nonalcoholic fatty liver disease and alcoholic liver disease^6,7^. Recent studies have suggested that cholestasis triggers the ER stress response^8–10^. Unresolved ER stress can contribute to the development of liver fibrosis^7^. The NLR family pyrin domain-containing 3 (NLRP3) inflammasome is a multiprotein complex composed of NLRP3, apoptosis-associated speck-like protein (ASC), and caspase-1^11^. The inflammasome results in the activation of caspase-1, leading to the cleavage of inactive pro-IL-1β into its biologically active form and the cleavage of gasdermin D (GSDMD) to mediate pyroptosis^12–14^. Increasing evidence shows that the NLRP3 inflammasome is activated in cholestatic liver injury in mice^15,16^.

Sestrins are a highly conserved protein family composed of Sestrin1, Sestrin2, and Sestrin3 in mammals. Sestrin expression can be upregulated in response to multiple cellular stresses, including DNA damage, oxidative stress, and hypoxia^17–19^. Sestrins are required for maintaining cellular homeostasis and suppressing age- and obesity-related pathologies by attenuating reactive oxygen species formation and mammalian target of rapamycin complex 1 (mTORC1) activation^7,19–21^. Sestrin2 expression is induced through the activation of p53 in response to genotoxic and oxidative stresses^22,23^, whereas the induction of Sestrin2 under hypoxia and ER stress is p53-independent^7,20,24^. In response to some stresses, Sestrin2 suppresses the activation of mTORC1 via AMP-activated protein kinase (AMPK)-tuberous sclerosis complex (TSC2)- or a Rag-dependent pathway^22,25,26^. Although a few studies, including ours, have documented the roles of Sestrin2 in the pathophysiology of liver diseases^7,19,27,28^, the relevance of hepatic Sestrin2 in cholestatic liver injury remains unexplored.

In this study, for the first time, we addressed these questions in an in vitro model of cholestasis using a bile acid-treated human hepatoma HepG2 cell line and in vivo using a bile-duct ligation (BDL) experimental model of cholestasis with Sestrin2 wild-type (*Sesn2*^+/+^) and knockout (*Sesn2*^−/−^) mice. We investigated the role of Sestrin2 induction in both bile acid-treated HepG2 cells and BDL mouse livers, as well as the molecular mechanisms by which Sestrin2 regulates cholestatic liver injury. Our results suggest that hepatic Sestrin2 expression is mediated by activating transcription factor 4 (ATF4) and CCAAT/enhancer-binding protein-β (C/EBP-β) upon cholestasis or bile acid treatment and correlates with ER stress responses. Sestrin2 deficiency promoted hepatic ER stress during cholestasis and exacerbated cholestasis-induced liver fibrosis and hepatocellular apoptosis. We also observed that cholestasis-induced Sestrin2 attenuates ER stress via the AMPK/mTORC1-dependent pathway. Furthermore, we found that Sestrin2 was involved in NLRP3-dependent pyroptosis in the context of cholestatic liver injury. Thus, our study identifies Sestrin2 as a potential therapeutic target for cholestatic liver diseases.

## Materials and methods

### Reagents

Immunoblotting was performed using antibodies against human Sestrin2 (Proteintech Group, USA), mouse Sestrin2 (Dr. Jun Hee Lee, University of Michigan); cleaved caspase-3, phospho-eIF2α, PERK, phospho-p70 S6 kinase, p70 S6 kinase, phospho-S6 ribosomal protein, S6 ribosomal protein, phospho-AMPK, AMPK, TSC2, NLRP3 (Cell Signaling Technology, USA), ATF4, C/EBP-β, eIF2α, caspase-1, ASC (Santa Cruz Biotechnology, USA); GSDMD (Abcam), GAPDH (Aviva Systems Biology, USA), and β-actin (Developmental Studies Hybridoma Bank, University of Iowa). Chenodeoxycholic acid (CDCA) and cholic acid (CA) were purchased from Sigma–Aldrich (USA). Rapamycin and AICAR were purchased from LC Laboratories (USA).

### Mice

Male, 6–8-week-old wild-type C57BL/6 mice were purchased from Samtako (Korea). *Sesn2*^*−/−*^ mice on a C57BL/6 background were kindly provided by Dr. Seo Goo Rhee (Yonsei University, Korea). All mice were maintained in a controlled temperature (20–25 °C) and humidity (50 ± 5%) environment with a 12 h light/dark cycle and free access to food and drinking water. All animal studies were conducted in accordance with the Guidelines for the Care and Use of Laboratory Animals of the National Institutes of Health and approved by the Animal Ethics Committee of Konyang University (P-20-37-A-01).

### Bile duct ligation procedure

Experiments were performed on 8-week-old male wild-type mice or 8-week-old male *Sesn2*^*−/−*^ mice (weighing 25–30 g) and corresponding wild-type controls that were subjected to sham surgery or BDL. The mice were anesthetized by an intraperitoneal injection of 100 mg/kg ketamine and 10 mg/kg xylazine. The mice underwent median laparotomies, and their common bile ducts were ligated twice with 4-0 silk sutures. Sham operations were performed on mice through an incision but without ligation of the common bile duct. The mice were allowed to recover from anesthesia and surgery under a red warming lamp and were housed singly. The mice were euthanized 3 days later to obtain liver samples. Liver samples were partially embedded in paraffin for histology or snap-frozen for molecular biology and biochemical analyses. Blood samples were collected to determine alanine aminotransferase (ALT), aspartate aminotransferase (AST), and alkaline phosphatase (ALP) activities. Serum levels of ALT, AST, and ALP were measured using ALT, AST, and ALP activity assay kits, respectively (BioVision, USA).

### Cell culture and treatments

The human hepatoma cell line HepG2 was cultured in Dulbecco’s modified Eagle’s medium (DMEM, Welgene, Korea) supplemented with 10% fetal bovine serum (FBS, Welgene) and 100 U/mL penicillin–streptomycin (Welgene). All cultures were maintained in a humidified 5% CO_2_ atmosphere at 37 °C. For bile acid treatment, cells were incubated in the presence of CA or CDCA as described previously^5^. Identical volumes of dimethyl sulfoxide (DMSO) were used as vehicle controls. When indicated, the cells were incubated with the mTOR inhibitor rapamycin or the AMPK activator AICAR.

### Plasmids and virus production

HEK293T cells were transfected with the following lentiviral constructs with the packaging plasmids using a polyethylenimine reagent: sh-Luciferase (sh-Luc), sh-Sestrin2, sh-p53, sh-TSC2, sh-C/EBP-β, cytomegalovirus promoter-green fluorescence protein (CMV-GFP), CMV-Flag-hSestrin2 constructs (kindly provided by Andrei V. Budanov, Trinity College Dublin)^7^, dominant-negative inositol-requiring enzyme 1α (IRE1α-DN), GFP constructs (Addgene, USA), sh-Luc, sh-ATF4, and sh-PERK constructs (Sigma–Aldrich). Lentiviral supernatants were collected and filtered 48 and 72 h after transfection. HepG2 cells were incubated for 2 days with the lentiviral medium in the presence of 4 µg/mL polybrene.

### Immunoblotting

Liver tissues and HepG2 cells were lysed in ice-cold radioimmunoprecipitation assay buffer containing a cOmplete^TM^ protease inhibitor cocktail (Roche, Switzerland). Lysates were incubated for 20 min on ice and centrifuged at 18,000 × *g* for 15 min at 4 °C. Protein concentrations were measured using the bicinchoninic acid protein assay (Pierce; Thermo Fisher Scientific, USA). Lysates were boiled in 1× SDS Laemmli sample buffer for 5 min. Proteins were separated by SDS-polyacrylamide gel electrophoresis and transferred to polyvinylidene fluoride membranes (Millipore, USA), which were probed with primary antibodies against Sestrin2, p-eIF2α, eIF2α, PERK, p-p70S6K, p70S6K, p-S6, S6, TSC2, ATF4, C/EBP-β, AMPK, NLRP3, ASC, Caspase1, GSDMD, GAPDH, and β-actin. After incubation with horseradish peroxidase-conjugated secondary antibodies, chemiluminescence was detected using the Fusion Solo System (Vilber Lourmat, France). Densitometric analysis of the blots was performed using ImageJ software (National Institutes of Health, USA), and the background was removed for each band.

### Luciferase reporter assays

Sestrin2 promoter constructs were generated by PCR amplification of the human Sestrin2 promoter (−1086 to −30 relative to the transcription start site) and subcloned into the pGL3-basic vector (Promega, USA) containing a firefly luciferase gene. HepG2 cells were transiently transfected with the pGL3-Sestrin2 plasmid (and pRL-TK plasmid with the Renilla luciferase gene as a control) using polyethylenimine. Forty-eight hours after transfection, the cells were treated with CDCA or CA for 9 h. The activities of both luciferases were measured using the Dual-Luciferase Reporter System (Promega) according to the manufacturer’s instructions. Luminescence was measured using a GloMax 20/20 luminometer (Promega). Relative luciferase activity was normalized to the firefly luminescence/Renilla luminescence ratio for each well.

### Quantitative real-time PCR

Total RNA was extracted from liver tissues and HepG2 cells using TRIzol reagent (Takara, Japan) according to the manufacturer’s instructions. Complementary DNA was synthesized using Moloney-murine leukemia virus reverse transcriptase (MMLV-RT, Promega) and random hexamer primers (BioFact, Korea). Quantitative real-time reverse transcription-PCR was performed in triplicate with SYBR green real-time PCR master mix reagent (Biofact) using the QuantStudio 3 Real-time PCR System (Life Technologies Inc., USA). Relative mRNA expression was calculated using comparative threshold cycle (C_t_) values normalized to mouse cyclophilin A expression. The following primers were used: human *SESN1*: forward (fwd) 5’-GCATGTTCCAACATTTCGTG-3’, reverse (rev) 5’-TCCCACATCTGGATAAAGGC-3’; human and mouse *SESN2*: fwd 5’-TAGCCTGCAGCCTCACCTAT-3’, rev 5’-TATCTGATGCCAAAGACGCA-3’; human *SESN3*: fwd 5’-ATGCTTTGGCAAGCTTTGTT-3’, rev 5’-GCAAGATCACAAACGCAGAA-3’; human *TGFB1*: fwd 5’- GGCCAGATCCTGTCCAAGC-3’, rev 5’-GTGGGTTTCCACCATTAGCAC-3’; human *CYP7A1*: fwd 5’-GAGAAGGCAAACGGGTGAAC-3’, rev 5’-GGATTGGCACCAAATTGCAGA-3’; human *Cyclophilin A*, fwd 5’-GCAAAGTGAAAGAAGGCATGAA-3’, rev 5’-CCATTCCTGGACCCAAAGC-3’; mouse *Sesn1*: fwd 5’-GGACGAGGAACTTGGAATCA-3’, rev 5’-ATGCATCTGTGCGTCTTCAC-3’; mouse *Sesn3*: fwd 5’-CATGCGTTTCCTCACTCAGA-3’, rev 5’-GGCAAAGTCTTCGTACCCAA-3’; mouse *Acta2*: fwd 5’-ACTGGGACGACATGGAAAAG-3’, rev 5’-GTTCAGTGGTGCCTCTGTCA-3’; mouse *Col1a*: fwd 5’-GCTCCTCTTAGGGGCCACT-3’, rev 5’-CCACGTCTCACCATTGGGG-3’; mouse *Tgfb1*: fwd 5’-CTCCCGTGGCTTCTAGTGC-3’, rev 5’-GCCTTAGTTTGGACAGGATCTG-3’; mouse *Cyp7a1*: fwd 5’-GGGATTGCTGTGGTAGTGAGC-3’, rev 5’-GGTATGGAATCAACCCGTTGTC-3’; mouse *Nlrp3*: fwd 5’-AGCCTTCCAGGATCCTCTTC-3’, rev 5’-CTTGGGCAGCAGTTTCTTTC-3’; mouse *Casp1*: fwd 5’-AGATGGCACATTTCCAGGAC-3’, rev 5’-GATCCTCCAGCAGCAACTTC-3’; mouse *Asc*: fwd 5’-GAAGCTGCTGACAGTGCAAC-3’, rev 5’-GCCACAGCTCCAGACTCTTC-3’; mouse *Il-1β*: fwd 5’-TCTTTGAAGTTGACGGACCC-3’, rev 5’- TGAGTGATACTGCCTGCCTG-3’; and mouse *cyclophilin A*, fwd 5’-GAGCTGTTTGCAGACAAAGTTC-3’, rev 5’-CCCTGGCACATGAATCCTGG-3’.

### Immunocytochemistry

HepG2 cells were grown on coverslips, rinsed with PBS, and fixed with 4% paraformaldehyde (pH 7.4) for 15 min at room temperature (18–22 °C). HepG2 cells were blocked in blocking solution for 1 h at room temperature and incubated with anti-cleaved caspase-3 (1:800), anti-PDI (1:400), or anti-BiP (1:400) antibodies overnight at 4 °C in a humidified chamber. After being washed, the cells were incubated with Alexa Fluor-conjugated secondary antibodies (1:500, Invitrogen, USA). Coverslips were mounted with ProLong Gold antifade reagent with 4-6-diamidino-2-phenylindole (Invitrogen). Fluorescent images were obtained using a laser scanning confocal microscope (LSM 700, Carl Zeiss, Germany) or an epifluorescence-equipped microscope (DM2500, Leica, Germany) and processed using the ImageJ software (NIH).

### Lipid peroxidation assay

The levels of malondialdehyde, the end product of lipid peroxidation, in cell lysates were measured using a lipid peroxidation assay kit (DOGEN, Korea) according to the standard protocol of the manufacturer. The absorbance was measured in an Epoch 2 microplate reader (Bio-Tek Instruments, USA) at 540 nm.

### Lactate dehydrogenase (LDH) cell death assay

After HepG2 cells were treated with bile acids, the culture supernatants were collected and used for cytotoxicity analysis using the Quanti-LDH™ Cytotoxicity Assay Kit (BioMAX, Korea) according to the manufacturer’s instructions. The absorbance was measured at 450 nm using an Epoch 2 microplate reader (Bio-Tek Instruments).

### Histology

Liver tissues were fixed in 10% neutral buffered formalin for 24 h, dehydrated, and embedded in paraffin. Section (5 μm) of the embedded tissue was prepared and stained with hematoxylin and eosin (H&E). For immunohistochemistry, paraffin-embedded sections were deparaffinized, rehydrated, and subjected to antigen retrieval. Endogenous peroxidase was quenched using 3% hydrogen peroxide. After nonspecific antigens were blocked, the sections were incubated with anti-cleaved caspase-3 or anti-PDI antibodies overnight at 4 °C, followed by incubation with biotinylated secondary antibodies (Vector Laboratories, USA). Antibodies were visualized with streptavidin-HRP (BD Biosciences, USA) using diaminobenzidine (Sigma–Aldrich). Hematoxylin was used to visualize nuclei. For Sirius red staining, the sections were incubated in 0.1% picrosirius red for 1 h. After being washed twice in acidified water, the sections were dehydrated with 100% ethanol and coverslipped. Samples were analyzed under a light microscope (Leica).

### Statistical analysis

The results are presented as the means ± standard error of the mean (SEM). The data presented in the figures are representative of at least three independent experiments unless otherwise stated. The significance of differences between the two experimental groups was determined using a two-tailed Student’s *t* test. Multiple comparisons were conducted with two-way analysis of variance followed by Tukey’s or Benjamini, Krieger, and Yekutieli’s post hoc tests. Differences were considered statistically significant at *p* < 0.05 (*p < 0.05; **p < 0.01; ***p < 0.001).

## Results

### Sestrin2 expression is upregulated in cholestatic livers

**Fig. 1:**
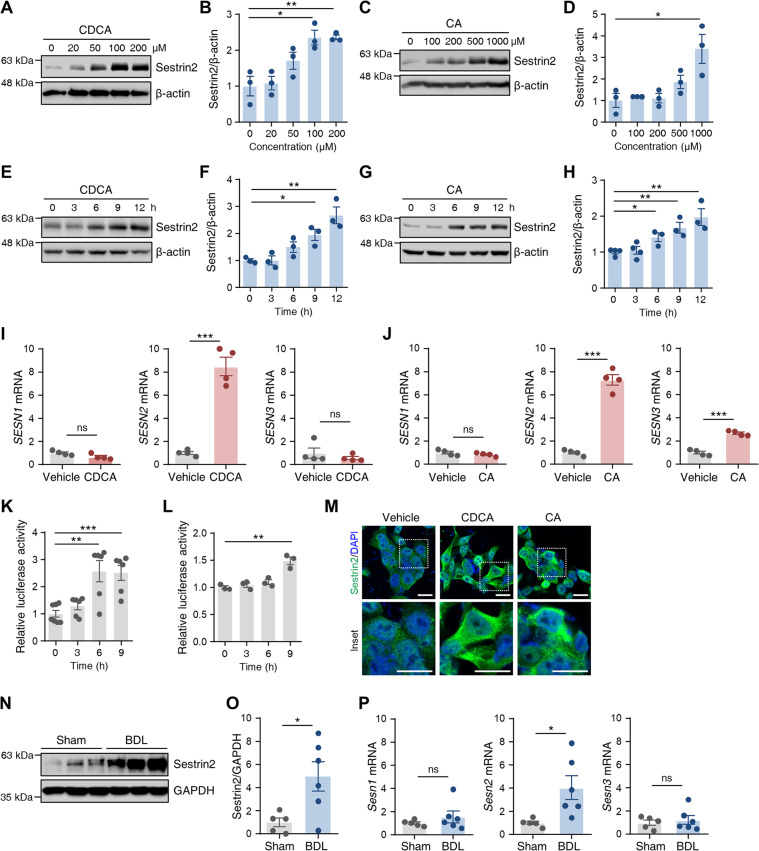
Sestrin2 is upregulated in bile acid-treated HepG2 cells and in BDL mouse livers. **A**–**D** HepG2 cells were treated with CDCA (20–200 µM) or CA (100–1000 µM) for 9 h, and the lysates were immunoblotted with anti-Sestrin2 antibodies (*n* = 3). **E**–**H** HepG2 cells were treated with 200 µM CDCA or 750 µM CA for the indicated times, and the lysates were immunoblotted with anti-Sestrin2 antibodies (*n* = 3–4). β-Actin served as a loading control. Band intensities were quantified and normalized to the β-actin values. **I**, **J** qRT–PCR analysis of *SESN1*, *SESN2*, and *SESN3* mRNA levels in HepG2 cells treated with 200 µM CDCA (**A**) or 750 µM CA (**B**) for 12 h (*n* = 4). **K**, **L** HepG2 cells were transfected with firefly luciferase reporter constructs containing a *SESN2* promoter sequence. At 48 h after transfection, the cells were treated with 200 µM CDCA (**K**) or 750 µM CA (**L**) for the indicated times (*n* = 3–6). Firefly luciferase activities were measured and normalized to Renilla luciferase activities. (**M**) Immunofluorescence staining of Sestrin2 (green) in HepG2 cells treated with 200 µM CDCA or 1000 µM CA for 9 h. Nuclei were stained with DAPI (blue). Scale bars, 20 μm. **N**–**P** Liver tissues were collected from mice 3 days after sham or BDL surgery (*n* = 5–6 mice per group) and analyzed by immunoblotting (**N**, **O**) and qRT–PCR (**P**). GAPDH served as a loading control. The data are representative of one (**N**–**P**) or three (**A**–**M**) independent experiments. *p < 0.05; **p < 0.01; ***p < 0.001; ns not significant (Student’s *t* test).

We evaluated the protein expression of Sestrin2 in HepG2 cells that were stimulated with the major components of bile acids: CDCA and CA. Immunoblot analysis showed that CDCA and CA increased Sestrin2 expression in a dose-dependent manner (Figs. 1A–D). The time course of Sestrin2 expression was further studied by treating the cells with CDCA (200 µM) or CA (750 µM)^5,29^, both of which significantly increased Sestrin2 expression in a time-dependent manner (Fig. 1E–H). Consistent with the protein levels, the mRNA expression of Sestrin2 was markedly elevated in CDCA- and CA-treated cells, while Sestrin1 and Sestrin3 mRNA levels were not affected or slightly increased (Fig. 1I, J). To assess the regulation of Sestrin2 transcription in CDCA- and CA-treated HepG2 cells, relative luciferase activity was measured. We found that expression was significantly higher in the CDCA- and CA-treated cells than in untreated cells (Fig. 1K, L). Correspondingly, the expression of Sestrin2 was increased in the cytoplasm of CDCA- and CA-treated cells (Fig. 1M). To measure the protein and mRNA expression levels of Sestrin2 in cholestatic livers, mice were subjected to BDL or sham operations, and liver tissues were collected 3 days after surgery. Immunoblot analysis showed that Sestrin2 protein expression levels were significantly elevated in the livers of BDL mice relative to those of sham controls (Fig. 1N, O). Consistent with the protein expression data, qRT–PCR showed significantly higher expression of Sestrin2 mRNA in livers 3 days after BDL than in the sham-operated group, while Sestrin1 and Sestrin3 mRNA levels were not affected (Fig. 1P). Collectively, these results suggest that the retention of bile acids increases hepatic Sestrin2 expression levels.

### Sestrin2 deficiency exacerbates cholestatic liver injury in bile duct-ligated mice

**Fig. 2:**
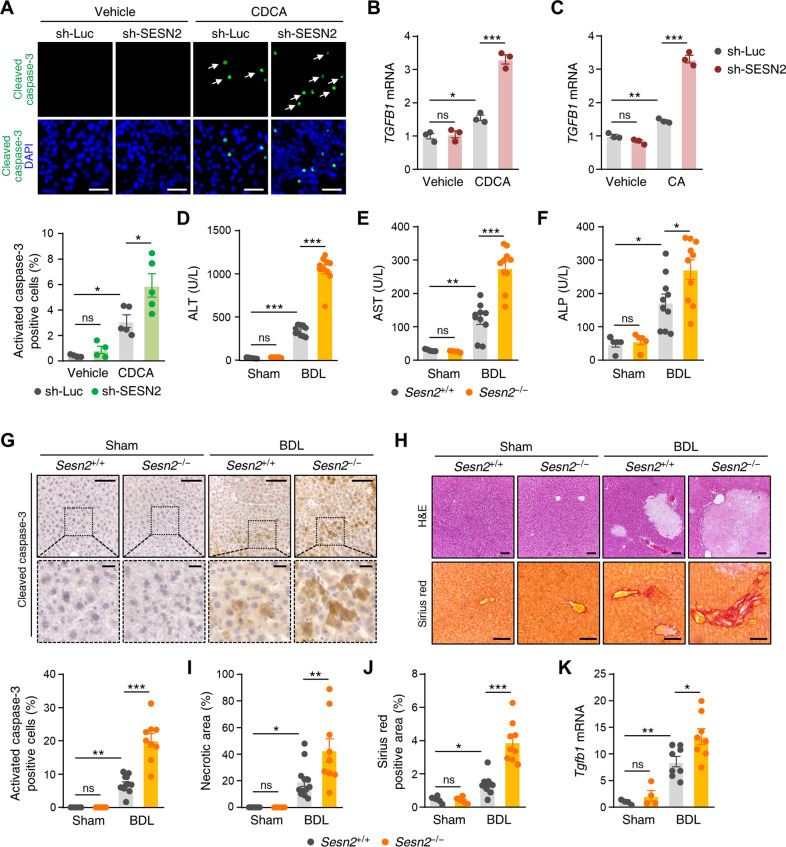
Sestrin2 deficiency exacerbates cholestatic liver injury. **A** Immunofluorescence staining of cleaved caspase-3 (green, arrows) in HepG2 cells infected with lentiviruses expressing shRNAs targeting luciferase (sh-Luc) or Sestrin2 (sh-SESN2) and treated with 150 µM CDCA for 12 h (*n* = 4–5). Nuclei were counterstained with DAPI (blue). **B**, **C** qRT–PCR analysis of *TGFB1* mRNA levels in HepG2 cells infected with sh-Luc or sh-SESN2 and treated with 200 µM CDCA or 750 µM CA for 12 h (*n* = 3). **D**–**F** Serum ALT, AST, and ALP levels in *Sesn2*^+/+^ Sham, *Sesn2*^+/+^ BDL, *Sesn2*^−/−^ Sham, and *Sesn2*^−/−^ BDL mice (*n* = 5-10 mice per group). **G**–**J** *Sesn2*^+/+^ and *Sesn2*^−/−^ mice were subjected to sham or bile duct ligation (BDL) for 3 days (*n* = 5–12 mice per group). **G** Immunohistochemical analysis of cleaved caspase-3 in liver tissues from *Sesn2*^+/+^ Sham, *Sesn2*^+/+^ BDL, *Sesn2*^−/−^ Sham, and *Sesn2*^−/−^ BDL mice. The boxed areas are magnified in the bottom panels. **H**, **I** H&E-stained liver sections from *Sesn2*^+/+^ Sham, *Sesn2*^+/+^ BDL, *Sesn2*^−/−^ Sham, and *Sesn2*^−/−^ BDL mice. Areas of necrosis were quantified. **H**, **J** Sirius red staining showing collagen fiber deposition in liver tissues from *Sesn2*^+/+^ Sham, *Sesn2*^+/+^ BDL, *Sesn2*^−/−^ Sham, and *Sesn2*^−/−^ BDL mice. **K** qRT–PCR analysis of *Tgfb1* mRNA levels in liver tissues from the indicated mice (*n* = 4–8 mice per group). Scale bars, 50 μm (a); 100 μm (**G**, **H**); 20 μm (**G**, insets). The data are representative of two (**D**–**K**) or three (**A**–**C**) independent experiments. *p < 0.05; **p < 0.01; ***p < 0.001; ns, not significant (two-way ANOVA, followed by Tukey’s (**A**–**F**, **K**) or Benjamini, Krieger, and Yekutieli’s (**I**, **J**) post hoc tests).

Because Sestrin2 is mainly elevated by stress and its expression was specifically stimulated in both bile acid-treated HepG2 cells and BDL mouse livers, we examined the effects of Sestrin2 deletion on bile acid-treated HepG2 cells and a murine model of cholestatic liver injury. Sestrin2 knockdown significantly increased the number of cleaved caspase-3-positive cells among CDCA-treated HepG2 cells (Fig. 2A). Hydrophobic bile acids can damage hepatocytes by increasing the generation of reactive oxygen species^30^. Lipid peroxidation levels were significantly higher in Sestrin2-knockdown cells than in control-knockdown cells (Supplementary Fig. 1a, b). Transforming growth factor β1 (*TGFB1*) mRNA levels were significantly more highly increased in Sestrin2-knockdown cells than in control-knockdown cells after treatment with bile acids (Fig. 2B, C). *Sesn2*^−/−^ BDL mice had significantly increased plasma ALT, AST, and ALP levels compared to *Sesn2*^+/+^ BDL mice (Fig. 2D–F). Correspondingly, immunohistochemical analysis of cleaved caspase-3 suggested that apoptotic cell death was significantly increased in *Sesn2*^−/−^ BDL livers compared with *Sesn2*^+/+^ BDL livers (Fig. 2G). Histological analysis revealed that more necrosis was present in *Sesn2*^−/−^ BDL livers than in *Sesn2*^+/+^ BDL livers (Fig. 2H, I). Liver fibrosis was measured by Sirius red staining and was significantly increased by BDL to a greater extent in *Sesn2*^−/−^ mice than in *Sesn2*^+/+^ mice (Fig. 2H, J). This finding correlated with a significantly increased ductular reaction after BDL in *Sesn2*^−/−^ mice compared to *Sesn2*^+/+^ mice (Supplementary Fig. 2). The mRNA expression of *Tgfb1*, α-SMA (*Acta2*), and collagen 1a (*Col1a*) was significantly higher in *Sesn2*^−/−^ BDL livers than in *Sesn2*^+/+^ BDL livers (Fig. 2K and Supplementary Fig. 3). We examined the effect of Sestrin2 deficiency on the gene expression of the bile acid-biosynthetic enzyme cytochrome-P450 7a1 (*Cyp7a1*) in bile acid-treated HepG2 cells and in the livers of BDL mice. *Cyp7a1* mRNA expression levels were not changed by Sestrin2 deficiency, although bile acids and BDL significantly reduced *Cyp7a1* mRNA expression (Supplementary Fig. 4a–c). These findings suggest that Sestrin2 is required to prevent liver damage during cholestasis.

### Bile acids upregulate Sestrin2 expression via an ATF4- and C/EBP-β-dependent mechanism

**Fig. 3:**
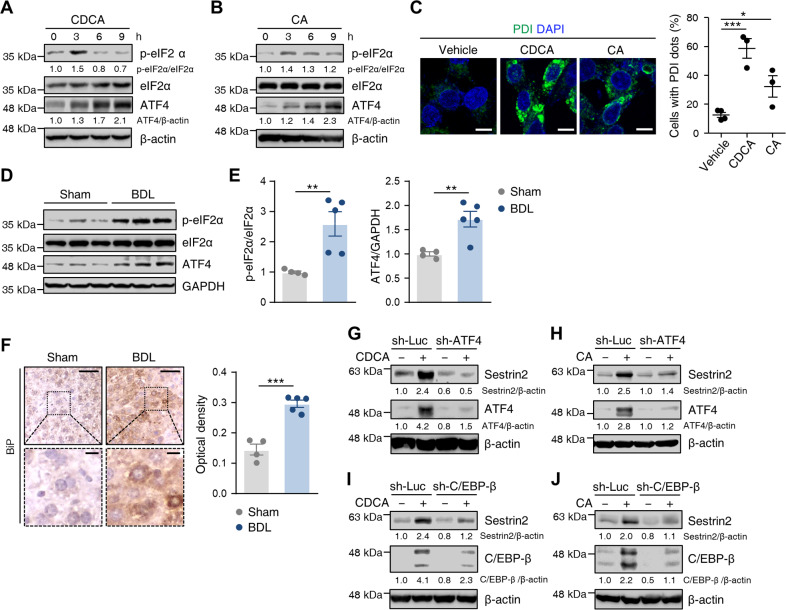
Bile acids upregulate Sestrin2 expression via an ATF4- and C/EBP-β-dependent mechanism. **A**, **B** HepG2 cells were treated with 200 µM CDCA or 750 µM CA for the indicated times. Cell lysates were immunoblotted with anti-p-eIF2α, anti-eIF2α, and anti-ATF4 antibodies. **C** Immunofluorescence staining of PDI (green) in HepG2 cells treated with 200 µM CDCA or 750 µM CA for 9 h (*n* = 3–4). Nuclei were stained with DAPI (blue). Scale bars, 10 μm. **D**, **E** Liver tissues were collected from mice 3 days after sham or BDL surgery (*n* = 4–5 mice per group) and analyzed by immunoblotting with the indicated antibodies. Band intensities were quantified and normalized to GAPDH or total protein intensities. **F** Immunohistochemical analysis of BiP in liver tissues from mice 3 days after sham or BDL surgery (*n* = 4–5 mice per group). The boxed areas are magnified in the bottom panels. Scale bars, 50 μm; 10 μm (insets). **G**, **H** HepG2 cells were infected with lentiviruses expressing shRNAs targeting luciferase (sh-Luc) or ATF4 (sh-ATF4) and treated with 200 µM CDCA or 750 µM CA for 9 h. Cell lysates were immunoblotted with anti-Sestrin2 and anti-ATF4 antibodies. **I**, **J** HepG2 cells were infected with lentiviral sh-Luc or sh-C/EBP-β and treated with 200 µM CDCA or 750 µM CA for 9 h. Cell lysates were immunoblotted with anti-Sestrin2 and anti-C/EBP-β antibodies. GAPDH or β-actin served as loading controls. Numbers below the immunoblot bands indicate fold changes normalized to the control band intensities. The data are representative of one (**D**–**F**) or at least three (**A**–**C**, **G**–**J**) independent experiments. *p < 0.05; **p < 0.01; ***p < 0.001 (Student’s *t* test).

Next, we explored the mechanism by which bile acids induce Sestrin2 expression. The effects of CDCA and CA on ER stress in HepG2 cells were examined by immunoblotting and immunocytochemistry. We found that the protein expression of the ER stress markers phosphorylated elF2α and ATF4 was stimulated by CDCA or CA in a time- and dose-dependent manner (Fig. 3A, B and Supplementary Fig. 5). Correspondingly, immunocytochemistry showed that the protein expression of the ER stress marker PDI was significantly elevated in CDCA- and CA-treated cells (Fig. 3C). To investigate the influence of cholestasis induced by BDL on ER stress, we measured the protein expression levels of ER stress markers in the livers of BDL mice. As expected, the expression of phosphorylated elF2α and ATF4 was markedly increased in the BDL liver (Fig. 3D, E). Immunohistochemical analysis of the ER stress marker BiP showed stronger expression in BDL livers than in sham control livers (Fig. 3F).

To determine whether ER stress-induced Sestrin2 expression in bile acid-treated HepG2 cells, we measured Sestrin2 expression after lentiviral knockdown of ATF4 and C/EBP-β. Knockdown of ATF4 abolished the induction of Sestrin2 expression in CDCA- and CA-treated cells (Fig. 3G, H). Knockdown of C/EBP-β also suppressed Sestrin2 expression in CDCA- and CA-treated cells (Fig. 3I, J). Furthermore, lentiviral knockdown of PERK, the primary regulator of ATF4 and C/EBP-β, also abrogated bile acid-induced Sestrin2 expression (Supplementary Fig. 6a, b). However, Sestrin2 protein levels did not change in CDCA-treated HepG2 cells after lentiviral knockdown of the ER stress sensor IRE1 (Supplementary Fig. 7a). Another regulator of Sestrin2 expression, p53, was not involved in Sestrin2 induction after exposure to CDCA (Supplementary Fig. 7b). Thus, these results suggest that bile acid-induced Sestrin2 expression is mediated by an ATF4- and C/EBP-β-dependent mechanism.

### Sestrin2 attenuates hepatic ER stress during cholestasis

**Fig. 4:**
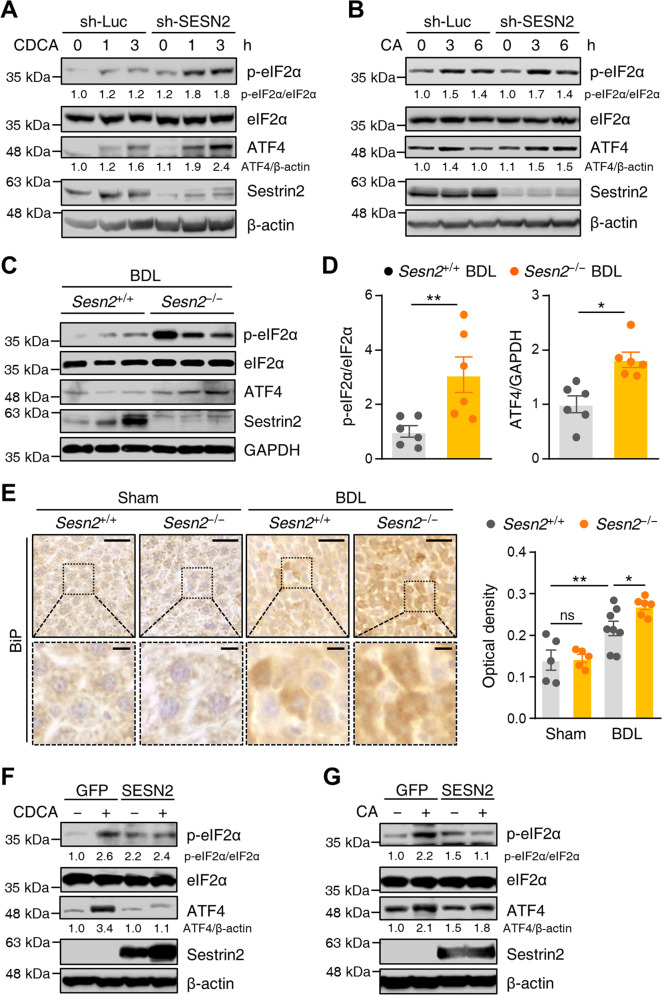
Sestrin2 deficiency exacerbates ER stress in cholestatic livers. **A**, **B** HepG2 cells were infected with lentiviruses expressing shRNAs targeting luciferase (sh-Luc) or Sestrin2 (sh-SESN2) and treated with 200 µM CDCA or 750 µM CA for the indicated times. Cell lysates were immunoblotted with anti-p-eIF2α, anti-eIF2α, anti-ATF4, and anti-Sestrin2 antibodies. β-Actin served as a loading control. Numbers below the immunoblot bands indicate fold changes normalized to the control band intensities. **C**, **D** Liver tissues were collected from *Sesn2*^+/+^ BDL and *Sesn2*^−/−^ BDL mice (*n* = 6 mice per group) and analyzed by immunoblotting with the indicated antibodies. GAPDH served as a loading control. Band intensities were quantified and normalized to GAPDH or total protein intensities. **E** Immunohistochemical analysis of BiP in liver tissues from *Sesn2*^+/+^ Sham, *Sesn2*^+/+^ BDL, *Sesn2*^−/−^ Sham, and *Sesn2*^−/−^ BDL mice (*n* = 5–8 mice per group). The boxed areas are magnified in the bottom panels. Scale bars, 50 μm; 10 μm (insets). **F**, **G** Immunoblot analysis of p-eIF2α, eIF2α, and ATF4 in HepG2 cells infected with lentiviruses expressing GFP as a control or Sestrin2 and treated with 200 µM CDCA or 750 µM CA for 3 h. β-Actin served as a loading control. Numbers below the immunoblot bands indicate fold changes normalized to the control band intensities. The data are representative of two (**C**–**E**) or three (**A**, **B**, **F**, **G**) independent experiments. *p < 0.05; **p < 0.01 (Student’s *t* test in **D** and two-way ANOVA, followed by Benjamini, Krieger, and Yekutieli’s post hoc test in **E**).

To further determine the role of Sestrin2 in bile acid-induced ER stress, we inhibited Sestrin2 expression in HepG2 cells using lentiviral vectors expressing shRNAs against human SESN2. Knockdown enhanced the levels of phosphorylated elF2α and ATF4 after the cells were treated with CDCA or CA (Fig. 4A, B). We next investigated whether Sestrin2 deficiency influenced hepatic ER stress in BDL mice. Immunoblot analysis of livers from *Sesn2*^−/−^ mice showed marked exacerbation of ER stress upon cholestasis, as evidenced by elevated levels of phosphorylated elF2α and ATF4 (Fig. 4C, D). Immunohistochemical analysis also suggested significant augmentation of hepatic ER stress in *Sesn2*^−/−^ BDL mice (Fig. 4E). To rule out the possibility that the elevated ER stress in Sestrin2-knockdown cells was caused by off-target effects, we performed a rescue experiment by ectopic expression of Sestrin2. Lentiviral expression of Sestrin2 resulted in a significant reduction in bile acid-induced ER stress compared to lentivirus-mediated expression of control GFP in Sestrin2-knockdown cells (Supplementary Fig. 8a, b). Furthermore, we investigated the effect of Sestrin2 overexpression on bile acid-induced ER stress in cells. Interestingly, CDCA- or CA-induced ER stress was suppressed by Sestrin2 overexpression (Fig. 4F, G). Taken together, these data suggest that Sestrin2 is required to attenuate bile acid-induced hepatic ER stress.

### Sestrin2 regulates AMPK/mTORC1 signaling during cholestasis

**Fig. 5:**
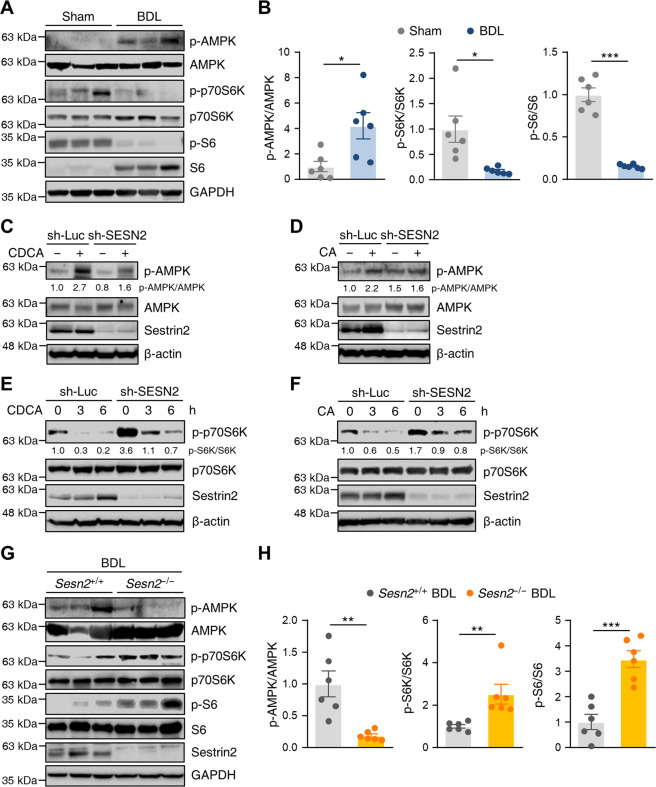
Sestrin2 regulates AMPK/mTORC1 signaling during cholestasis. **A**, **B** Liver tissues were collected from mice 3 days after sham or BDL surgery (*n* = 6 mice per group) and analyzed by immunoblotting with anti-p-AMPK, anti-AMPK, anti-p-p70S6K, anti-p70S6K, anti-p-S6, and anti-S6 antibodies. Band intensities were quantified and normalized to total protein intensities. **C**–**F** HepG2 cells were infected with lentiviruses expressing shRNAs targeting luciferase (sh-Luc) or Sestrin2 (sh-SESN2) and treated with 200 µM CDCA or 750 µM CA for the indicated hours. Cell lysates were immunoblotted with the indicated antibodies. β-Actin served as a loading control. Numbers below the immunoblot bands indicate the fold changes normalized to the control band intensities. **G**, **H** Liver tissues were collected from *Sesn2*^+/+^ BDL and *Sesn2*^−/−^ BDL mice (*n* = 6 mice per group) and analyzed by immunoblotting with the indicated antibodies. Band intensities were quantified and normalized to total protein intensities. The data are representative of two (**A**, **B**, **G**, **H**) or at least three (**C**–**F**) independent experiments. *p < 0.05; **p < 0.01; ***p < 0.001 (Student’s *t* test).

We have previously reported a role for Sestrin2 in the regulation of AMPK-mTORC1 in response to multiple stresses^7,20,21^. We therefore examined the protein expression of downstream targets of AMPK and mTORC1 in bile acid-treated HepG2 cells and the livers of BDL mice. Immunoblot analysis indicated that the phosphorylation of AMPK was increased, while the phosphorylation of p70S6K and ribosomal protein S6 levels were significantly downregulated in BDL mice compared to sham controls (Fig. 5A, B). CDCA and CA activated AMPK but suppressed the phosphorylation of p70S6K in HepG2 cells in a time-dependent manner (Supplementary Fig. 9a–d).

Next, we determined the regulatory effect of Sestrin2 deficiency on the AMPK-mTORC1 pathway in bile acid-treated HepG2 cells and the livers of BDL mice. Knockdown of Sestrin2 inhibited the phosphorylation of AMPK (Fig. 5C, D) and elevated the phosphorylation of p70S6K upon exposure to CDCA and CA (Fig. 5E, F). Correspondingly, the phosphorylation of AMPK was significantly lower in *Sesn2*^−/−^ BDL mice than in *Sesn2*^+/+^ BDL mice, and the phosphorylation of p70S6K and S6 were significantly higher in *Sesn2*^−/−^ BDL livers than in *Sesn2*^+/+^ BDL livers (Fig. 5G, H), indicating that bile acid-induced Sestrin2 negatively regulates mTORC1 activity during cholestasis.

### Sestrin2 attenuates bile acid-induced ER stress via an AMPK/mTORC1-dependent mechanism

**Fig. 6:**
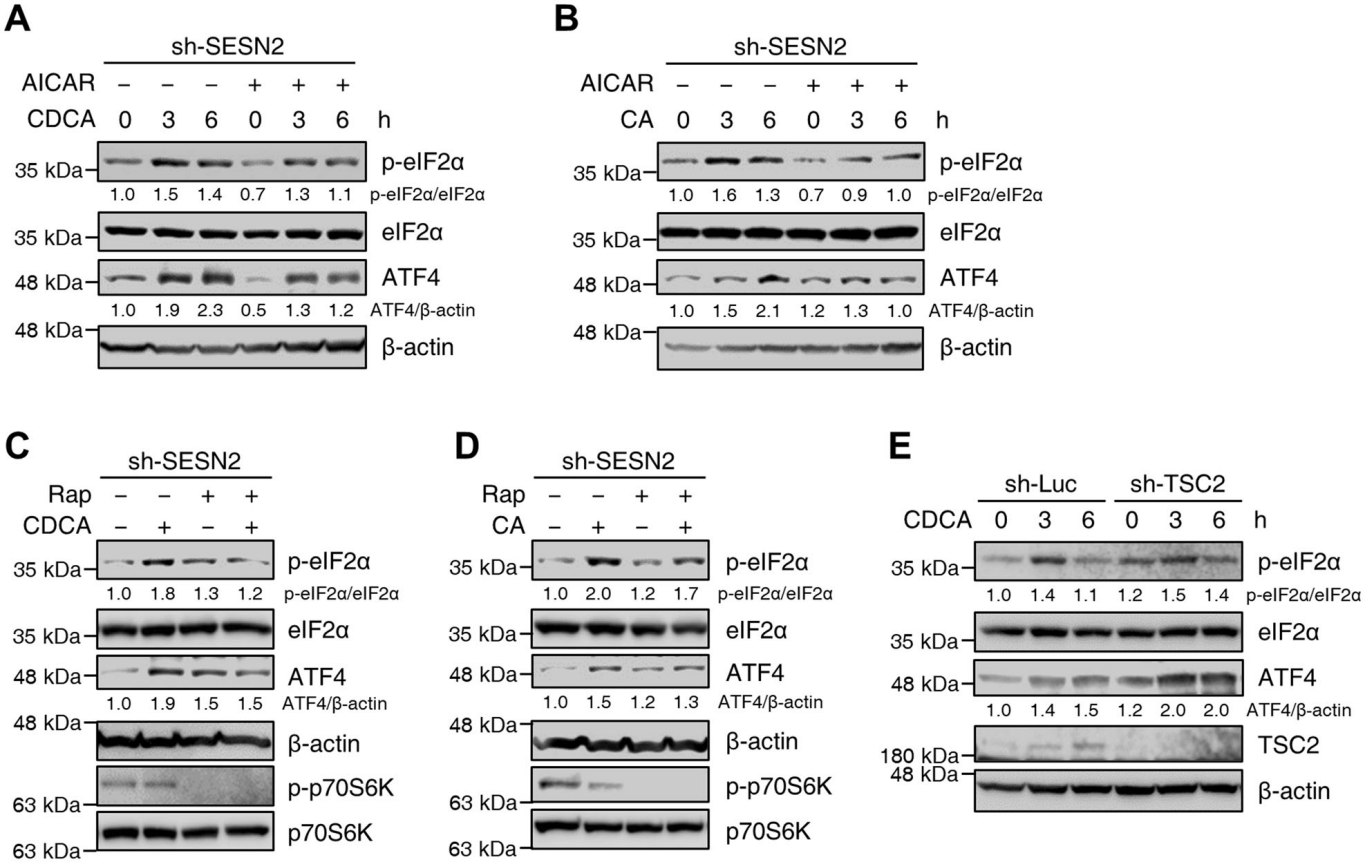
Sestrin2 attenuates bile acid-induced ER stress via an AMPK/mTORC1-dependent mechanism. **A**, **B** HepG2 cells were infected with sh-SESN2 lentivirus and treated with 200 µM CDCA or 750 µM CA for the indicated times in the presence or absence of 100 µM AICAR. Cell lysates were immunoblotted with the indicated antibodies. **C**, **D** HepG2 cells were infected with sh-SESN2 lentivirus and treated with 200 µM CDCA or 750 µM CA for 9 h in the presence or absence of 100 nM rapamycin (Rap). Cell lysates were immunoblotted with the indicated antibodies. **E** HepG2 cells were infected with sh-Luc or sh-TSC2 lentiviruses and treated with 200 µM CDCA for the indicated times. Cell lysates were immunoblotted with the indicated antibodies. β-Actin served as a loading control. Numbers below the immunoblot bands indicate the fold changes normalized to the control band intensities. The data are representative of at least three independent experiments.

To investigate whether AMPK activation in Sestrin2-deficient cells influences bile acid-induced ER stress, we treated Sestrin2-knockdown HepG2 cells with the AMPK activator AICAR and performed immunoblotting. Interestingly, treatment with AICAR abrogated the increased levels of phosphorylated elF2α and ATF4 in Sestrin2-deficient cells (Fig. 6A, B). We next examined whether treatment with rapamycin, a mTOR inhibitor, in Sestrin2-deficient cells influenced bile acid-induced ER stress. We observed suppression of the bile acid-induced increase in the levels of phosphorylated elF2α and ATF4 in Sestrin2-deficient cells (Fig. 6C, D). Next, we explored the effect of mTORC1 activation by TSC2-shRNA on bile acid-induced ER stress. We found that the phosphorylation of elF2α and ATF4 was significantly higher in TSC2-knockdown cells than in control-knockdown cells after treatment with CDCA (Fig. 6E). Thus, these results indicate that Sestrin2 attenuates ER stress during cholestasis in an AMPK/mTORC1-dependent manner.

### Sestrin2 deficiency exacerbates NLRP3 inflammasome-mediated pyroptosis in cholestatic livers

**Fig. 7:**
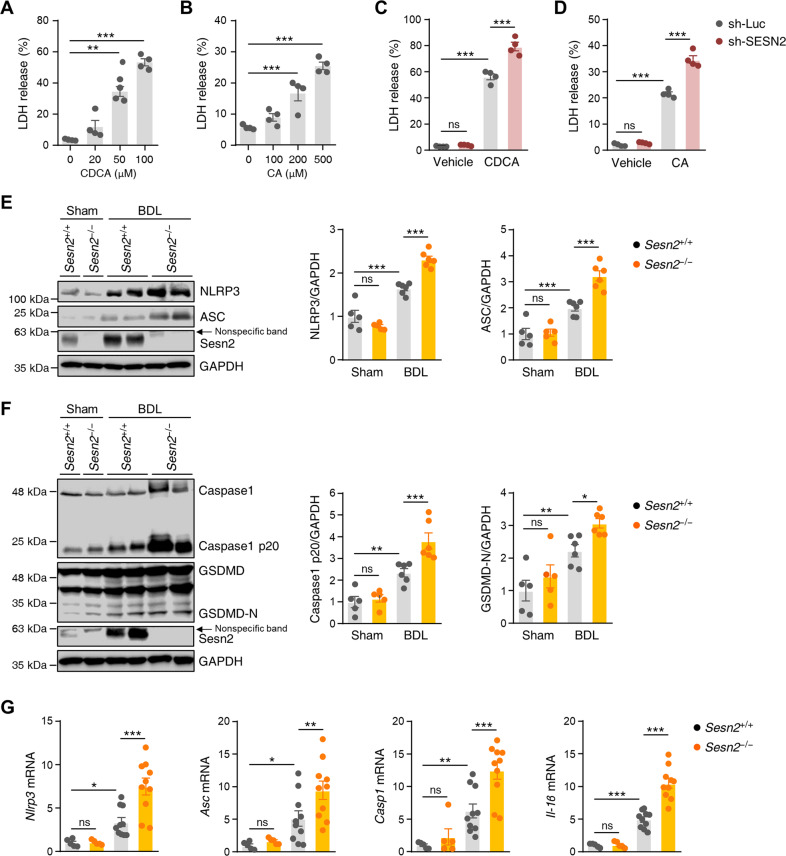
Sestrin2 deficiency exacerbates NLRP3 inflammasome-mediated pyroptosis in cholestatic livers. **A**, **B** HepG2 cells were treated with CDCA (20 to 100 µM) or CA (100 to 500 µM) for 24 h. The release of LDH was measured by an LDH assay kit. **C**, **D** HepG2 cells were infected with lentiviruses expressing shRNAs targeting luciferase (sh-Luc) or Sestrin2 (sh-SESN2) and treated with 100 µM CDCA or 500 µM CA for 24 h. The release of LDH was measured by an LDH assay kit. **E**, **F** Liver tissues were collected from *Sesn2*^+/+^ Sham, *Sesn2*^+/+^ BDL, *Sesn2*^−/−^ Sham, and *Sesn2*^−/−^ BDL mice (*n* = 5–6 mice per group) and analyzed by immunoblotting with the indicated antibodies. GAPDH served as a loading control. Band intensities were quantified and normalized to control band intensities. **G** qRT–PCR analysis of *Nlrp3*, *Asc*, *Casp1*, and *Il-1β* mRNA levels in liver tissues from the indicated mice (*n* = 5–10 mice per group). The data are representative of two (**E**–**G**) or three (**A**–**D**) independent experiments. *p < 0.05; **p < 0.01; ***p < 0.001; ns not significant (Student’s *t* test in **A**, **B** and two-way ANOVA, followed by Tukey’s (**C**, **D**) or Benjamini, Krieger, and Yekutieli’s (**E**–**G**) post hoc tests).

Emerging evidence suggests that NLRP3 inflammasome activation is involved in hepatocyte pyroptosis, liver inflammation, and fibrosis^31,32^. To evaluate whether bile acids affect pyroptotic cell death, the effects of CDCA and CA on LDH release in HepG2 cells were examined by LDH release assays. As shown in Fig. 7A, B, the release of LDH in cell supernatants was increased by CDCA or CA in a dose-dependent manner. Furthermore, we investigated whether Sestrin2 was involved in bile acid-induced pyroptosis. When cells were transduced with lentiviruses expressing Sestrin2-shRNA, bile acid-induced LDH secretion was significantly increased (Fig. 7C, D). We next determined whether Sestrin2 knockout alters NLRP3 inflammasome activation in the livers of BDL mice. Immunoblot analysis suggested that the protein expression levels of NLRP3 and ASC were significantly higher in *Sesn2*^−/−^ BDL livers than in *Sesn2*^+/+^ BDL livers (Fig. 7E). Correspondingly, *Sesn2*^−/−^ BDL mice had substantially increased protein expression levels of the pyroptosis markers GSDMD and caspase-1 compared to *Sesn2*^+/+^ BDL mice (Fig. 7F). Consistent with the protein expression data, qRT–PCR showed significantly higher mRNA expression of Nlrp3, Asc, Casp1, and Il-1β in *Sesn2*^−/−^ BDL livers than in *Sesn2*^+/+^ BDL livers (Fig. 7G). Overall, these results suggest that Sestrin2 can alleviate NLRP3 inflammasome-mediated pyroptosis in cholestatic livers.

## Discussion

**Fig. 8:**
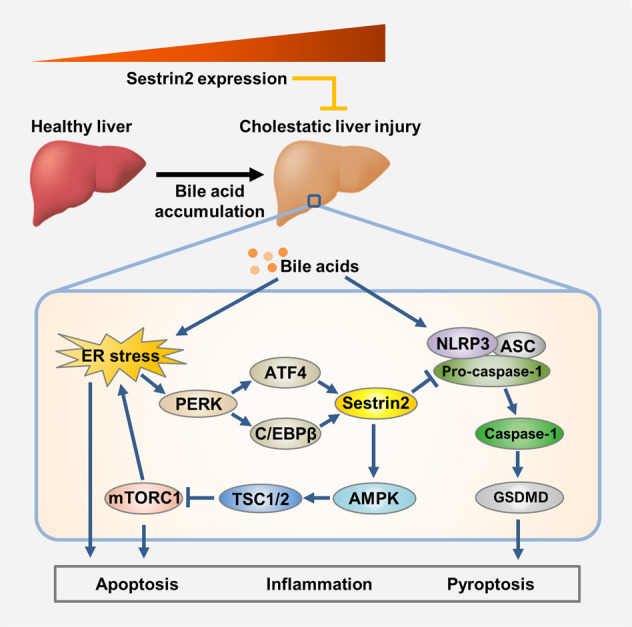
Schematic illustrating the protective effect and mechanism of Sestrin2 in cholestatic liver injury. Hepatic Sestrin2 expression is induced by ATF4 and C/EBP-β during cholestatic liver injury and thereby inhibits mTORC1 activity. Sestrin2 also suppresses NLRP3 inflammasome activation and subsequently relieves pyroptosis and inflammatory response in cholestatic livers.

Cholestasis causes the accumulation of bile acids in the liver, leading to hepatic injury. Although the upregulation of Sestrin2 is well documented in the pathophysiology of liver diseases^7,19,27,28^, there have been no studies investigating the regulation of Sestrin2 in the pathogenesis of bile acid-induced liver injury. Here, we observed increased transcription and translation of Sestrin2 in response to pathophysiological levels of bile acids, as evidenced by the in vitro and in vivo models of cholestasis. Activation of the unfolded protein response (UPR), a signaling cascade and transcriptional event induced by ER stress, has been associated with many metabolic and hepatic diseases, including obesity, fatty liver disease, and cholestatic liver disease^7–10^. Increasing evidence suggests that the induction of Sestrin2 expression is involved in metabolism and age-related diseases^7,19,20^. However, the role of Sestrin2 regulation in cholestatic liver injury remains elusive. In the present study, we showed that hepatic Sestrin2 expression was elevated by BDL in an ER stress-dependent manner. Furthermore, the loss of Sestrin2 increased hepatic vulnerability to cholestatic liver injury, as assessed by serum ALT levels, fibrosis, and apoptosis (Fig. 8), suggesting that Sestrin2 is involved in protecting against cholestatic liver disease.

Cholestasis causes hepatocellular bile acid retention, and excessive intrahepatic accumulation of bile acids may result in liver injury^33^. In vitro, the accumulation of toxic hydrophobic bile acids can result in hepatocyte apoptosis. Hepatic cell death, including necrosis and apoptosis, was induced in a BDL-induced mouse model of cholestasis, as previously described^5^. The upregulation of Sestrin2 expression has been shown to inhibit apoptotic cell death induced by multiple stresses, including ER stress, DNA damage, and oxidative stress^7,34^. However, the molecular links between bile acid-induced hepatic apoptosis and Sestrin2 expression are not yet understood. In our *Sesn2*^−/−^ mouse model, increases in necrotic areas and cleaved caspase-3-positive cells were observed after BDL. We also found that Sestrin2 knockdown caused an apparent increase in the number of cleaved caspase-3-positive cells in CDCA-treated HepG2 cells. ER stress and the accumulation of unfolded proteins in the ER release the levels of the ER stress sensor molecules IRE1, PERK, and ATF6, which regulate transcription, translation, and apoptosis^35^. Severe or prolonged ER stress is correlated with apoptosis in liver cells^36,37^. Our results clearly demonstrated severe ER stress in the livers of Sestrin2-deficient mice during cholestasis. Thus, our data suggest that Sestrin2 is intimately involved in cholestasis-induced hepatocellular apoptosis.

Although hepatic stellate cells are known to play an important role in hepatic fibrogenesis, hepatocytes, which are the major parenchymal cells in the liver, actively trigger profibrogenic responses. Apoptotic hepatocytes produce apoptotic bodies that are removed via phagocytosis by Kupffer cells, hepatic stellate cells, and hepatocytes^38^. Apoptotic body phagocytosis has also been reported to promote the secretion of chemokines and cytokines, such as TGF-β1, and trigger the activation of hepatic stellate cells, leading to fibrosis^39,40^. Because Sestrin2 is known to be expressed in hepatocytes, hepatic stellate cells, liver sinusoidal endothelial cells, and other immune cells^7,41^, it is possible that the observed effects of Sestrin2 deficiency on cholestatic liver fibrosis were mediated by factors released from nonhepatocytes. Given that hepatic stellate cell activation could be inhibited by Sestrin2 expression^41,42^, alternative explanations are possible in the context of Sestrin2-expressing nonhepatocytes. Enhanced TGF-β signaling is known to be associated with hepatic fibrosis in different animal models and human patients^43^. In addition, our results clearly demonstrate that Sestrin2 deficiency strongly upregulates TGF-β1 expression in both bile acid-treated HepG2 cells and BDL mouse livers. Therefore, these findings reinforce the notion that Sestrin2 also has a role in inhibiting fibrogenic and inflammatory cytokine production.

Hydrophobic bile acids are known to be cytotoxic to cells in the liver, especially hepatocytes, because of their detergent properties^44,45^. The intensities of cytotoxicity and ER stress induced by bile acids are generally dependent on the magnitude of their hydrophobicity^46^. Indeed, Adachi et al. demonstrated that bile acids caused ER stress and subsequent apoptosis in HepG2 cells in a hydrophobicity-dependent manner^47^. In many studies, cell culture models are often used to assess the direct cytotoxic effects of bile acids on hepatocytes^5,29,44,48^. We observed cytotoxicity when HepG2 cells were exposed to ≥50 µM CDCA or ≥200 µM CA. However, these concentrations are higher than the pathologically measured concentrations of bile acids^49^. To address this technical limitation, further investigation on the correlation between the cytotoxic effect of bile acids in vivo and in vitro will be needed.

Signaling by mTORC1 can stimulate nutrient-consuming anabolic processes, such as protein synthesis, through the phosphorylation of p70S6K and eIF4E-binding protein (4E-BP)^50^. Accumulating evidence has implicated the dysregulation of mTORC1 in the exacerbation of ER stress^7,51^. Consistent with these findings, we found that TSC2-deficient HepG2 cells were more susceptible to bile acid-induced ER stress than control cells. Lentiviral knockdown of TSC2 exacerbated ER stress, presumably because of mTOR hyperactivation-induced protein overproduction^7,50^. The occurrence of ER stress may be associated with an imbalance in protein synthesis and degradation. Our results clearly demonstrate that cholestasis-induced Sestrin2 expression suppresses mTOR activity by activating AMPK. The inhibitory effect of Sestrin2 on hepatic ER stress during cholestasis may be the result of protein translation inhibition through the AMPK/mTOR pathway. We found strong evidence that the inhibitory effect of Sestrin2 on ER stress is dependent on the AMPK/mTOR pathway by demonstrating that AICAR treatment of Sestrin2-deficient cells attenuated bile acid-induced ER stress, and rapamycin treatment of Sestrin2-deficient cells suppressed bile acid-induced ER stress. Taken together, our results support the hypothesis that the exacerbation of ER stress by defective AMPK/mTORC1 regulation is a major mechanism in the pathophysiology of cholestasis.

Sestrin2 can enhance autophagy by inhibiting the mTORC1 pathway under multiple pathological conditions^52,53^. Enhanced autophagy contributes to cellular protein homeostasis by maintaining protein quality control and can consequently alleviate ER stress^54,55^. Sestrin2 may be implicated in the suppression of cholestasis-induced ER stress through autophagy activation, although further experimental studies are needed to investigate the role of Sestrin2-mediated autophagy in attenuating cholestasis-induced liver injury.

NLRP3 inflammasome activation in hepatocytes not only mediates the chronic inflammatory response but can also induce hepatocyte pyroptosis and hepatic stellate cell activation, which then cause liver fibrosis and collagen deposition^31,56^. Wree et al. showed that the expression of a constitutively activated form of NLRP3 in hepatocytes causes pyroptotic cell death, which contributes to liver injury and fibrosis^31^. Similar to our findings, Frissen et al. revealed that NLRP3 inflammasome activation is associated with acute and chronic cholestatic liver injury^16^. Notably, Nlrp3-deficient mice have decreased liver injury and inflammation in chronic cholestasis. A recent study suggested that Sestrin2 deficiency-induced NLRP3 inflammasome activation and pyroptosis, which subsequently increased the mortality of septic mice^57^. Our results showed a similar effect, as both the NLRP3 inflammasome and pyroptosis were increased in *Sesn2*^−/−^ BDL mice. Other studies have demonstrated the interactions between ER stress and the NLRP3 inflammasome^58,59^. However, future investigation will be needed to explore the molecular mechanism underlying the regulation of NLRP3 inflammasome signaling by Sestrin2 during cholestatic liver injury.

In conclusion, this study provides evidence for the important role of Sestrin2 in attenuating bile acid-induced NLRP3 inflammasome activation and ER stress and attenuating cholestatic liver injury. Increased hepatic ER stress due to cholestasis is a major contributor to the upregulated expression of ATF4 and C/EBP-β, which mediate Sestrin2 induction. In addition, our study demonstrates that Sestrin2 regulates cholestasis-induced NLRP3 inflammasome activation and pyroptosis. Our study reveals the molecular mechanism underlying the beneficial effect of Sestrin2 on bile acid-induced apoptosis and pyroptosis, suggesting that Sestrin2 may be a therapeutic target for the treatment of the cholestatic liver injury.

## Supplementary information

Supplementary information
