# Supplementary material for: Sestrin2 protects against cholestatic liver injury by inhibiting endoplasmic reticulum stress and NLRP3 inflammasome-mediated pyroptosis
Source: Exp Mol Med. 2022 Mar 8;54(3):239–51. doi: 10.1038/s12276-022-00737-9 (PMC8980001; doi:10.1038/s12276-022-00737-9)
Supplement: Supplementary file 1 — Supplementary information [file 12276_2022_737_MOESM1_ESM.pdf]

Sestrin2 protects against cholestatic liver injury by inhibiting endoplasmic reticulum stress and NLRP3 inflammasome-mediated pyroptosis

Daewon Han, Haeil Kim, Soojin Kim, Qui Anh Le, Seung Yun Han, Jeongyun Bae, Hye Won Shin, Hyun-Goo Kang, Kyung Ho Han, Jongdae Shin, and Hwan-Woo Park

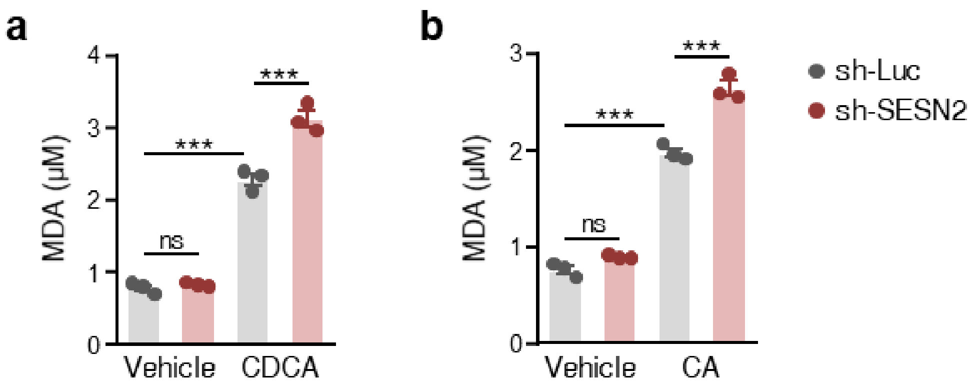

Supplementary Fig. 1. (a, b) Lipid peroxidation (MDA) levels in HepG2 cells infected with lentiviruses expressing shRNAs targeting luciferase (sh-Luc) or Sestrin2 (sh-SESN2) and treated with 200 μM CDCA or 750 μM CA for 12 h (n = 3). Data are representative of three independent experiments. ***p < 0.001; ns, not significant (Two-way ANOVA, followed by Tukey's post hoc tests).

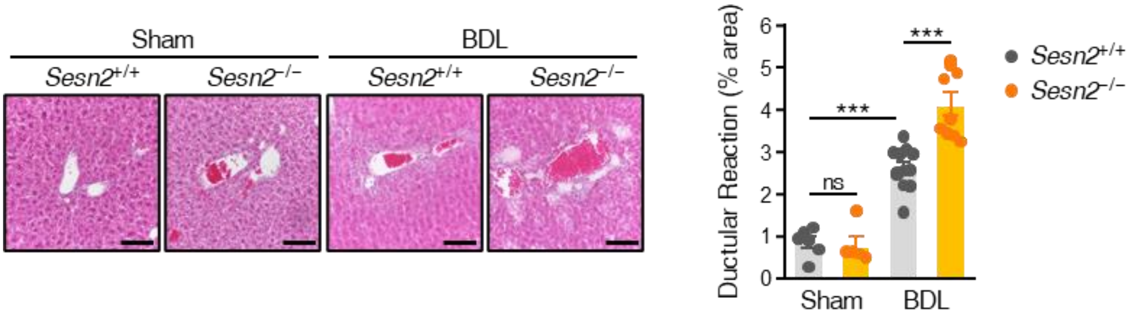

Supplementary Fig. 2. *Sesn2*$^{+/+}$ and *Sesn2*$^{-/-}$ mice were subjected to sham or bile duct ligation (BDL) for 3 days (n = 5-12 mice per group). H&E-stained liver sections from *Sesn2*$^{+/+}$ Sham, *Sesn2*$^{+/+}$ BDL, *Sesn2*$^{-/-}$ Sham, and *Sesn2*$^{-/-}$ BDL mice, displaying prominent ductular reaction. Areas of ductular reaction were quantified. Data are representative of two independent experiments. ***$p < 0.001$; ns, not significant (Two-way ANOVA, followed by Tukey's post hoc tests).

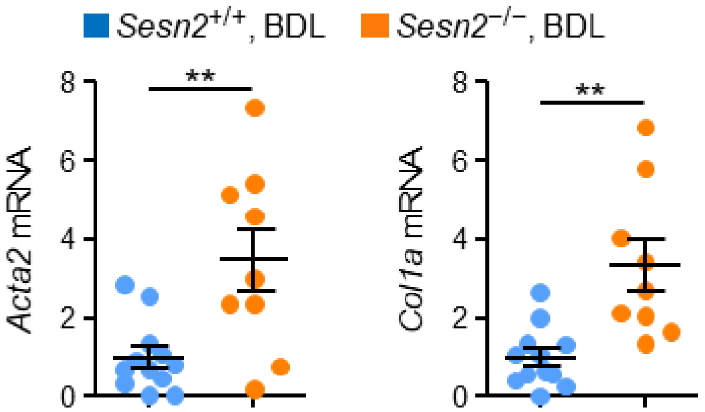

Supplementary Fig. 3. Liver tissues were collected from *Sesn2*$^{+/+}$ BDL and *Sesn2*$^{-/-}$ BDL mice (n = 9-12 mice per group). Relative mRNA expression levels of *Acta2* and *Col1a* were determined by qRT-PCR. Data are representative of two independent experiments. **p < 0.01 (Student's *t*-test).

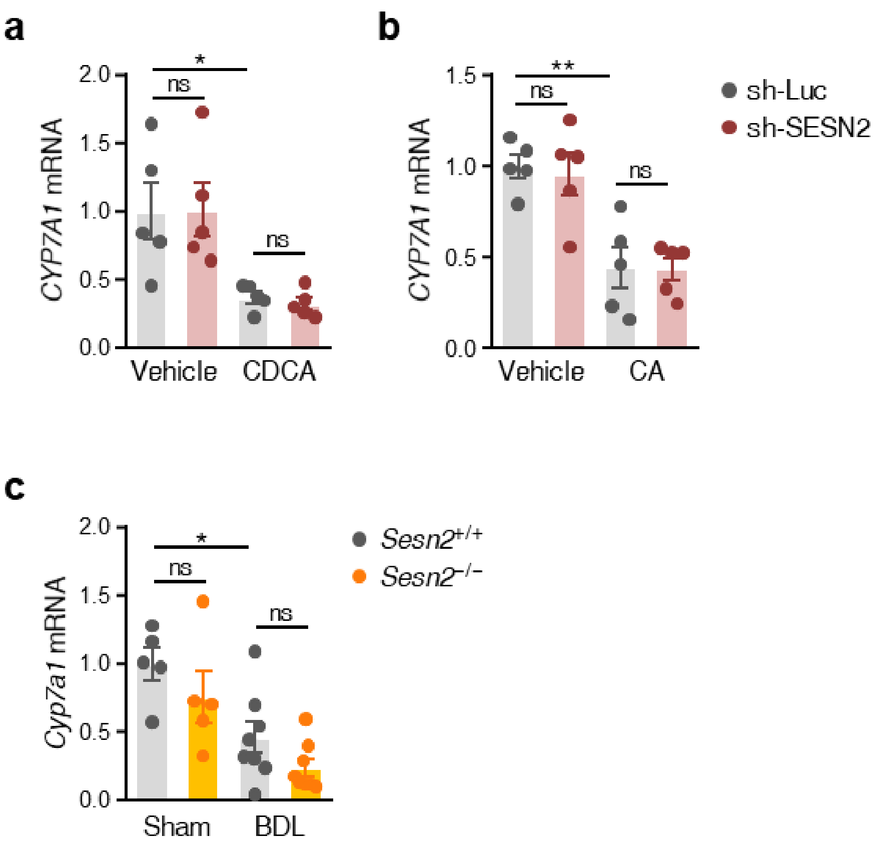

Supplementary Fig. 4. (a, b) qRT-PCR analysis of *CYP7A1* mRNA levels in HepG2 cells infected with sh-Luc or sh-SESN2 and treated with 200 µM CDCA or 750 µM CA for 12 h (n = 5). (c) qRT-PCR analysis of *Cyp7a1* mRNA levels in liver tissues from the indicated mice (n = 5-8 mice per group). Data are representative of two (c) or three (a, b) independent experiments. *p < 0.05; **p < 0.01; ns, not significant (Two-way ANOVA, followed by Tukey's post hoc tests).

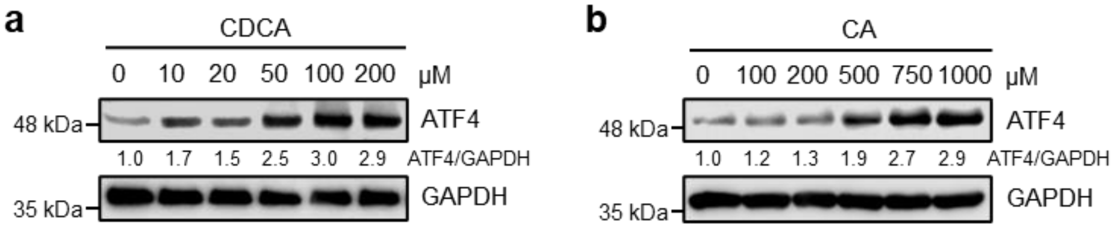

Supplementary Fig. 5. (a, b) HepG2 cells were treated with CDCA (10 to 200 μM) or CA (100 to 1000 μM) for 9 h. Cell lysates were immunoblotted with anti-ATF4 antibody. GAPDH served as a loading control. Numbers below immunoblot bands indicate the fold changes normalized to control band intensities. Results are representative of at least three independent experiments.

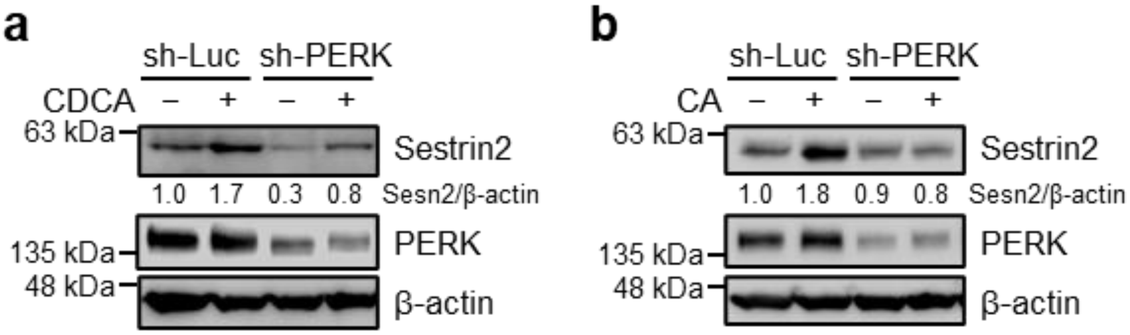

Supplementary Fig. 6. (a, b) HepG2 cells were infected with sh-Luc or sh-PERK, and treated with 200 μM CDCA or 750 μM CA for 9 h. Cell lysates were immunoblotted with anti-Sestrin2 and anti-PERK antibodies. β-actin served as a loading control. Numbers below immunoblot bands indicate the fold changes normalized to control band intensities. Results are representative of at least three independent experiments.

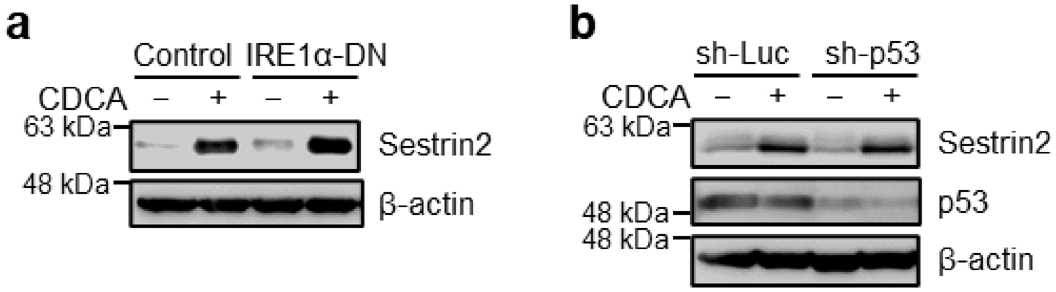

Supplementary Fig. 7. (a) HepG2 cells were infected with lentiviruses expressing GFP (Control) or a dominant-negative form of IRE1α (IRE1α-DN) and treated with 200 μM CDCA for 9 h. Cell lysates were immunoblotted with anti-Sestrin2 antibody. (b) HepG2 cells were infected with lentiviral sh-Luc or sh-p53 and treated with 200 μM CDCA for 9 h. Cell lysates were immunoblotted with anti-Sestrin2 and anti-p53 antibodies. β-actin served as a loading control. Results are representative of at least three independent experiments.

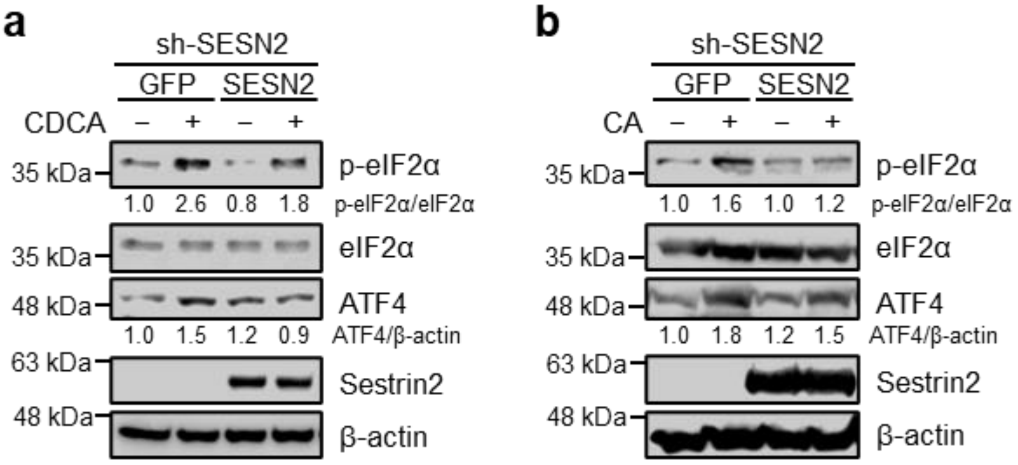

Supplementary Fig. 8. (a, b) Sestrin2 knockdown HepG2 cells were infected with lentiviruses expressing GFP as control or Sestrin2 and treated with 200 μM CDCA or 750 μM CA for 3 h. Cell lysates were immunoblotted with anti-p-eIF2α, anti-eIF2α, anti-ATF4, and anti-Sestrin2 antibodies. β-actin served as a loading control. Numbers below immunoblot bands indicate the fold changes normalized to control band intensities. Results are representative of at least three independent experiments.

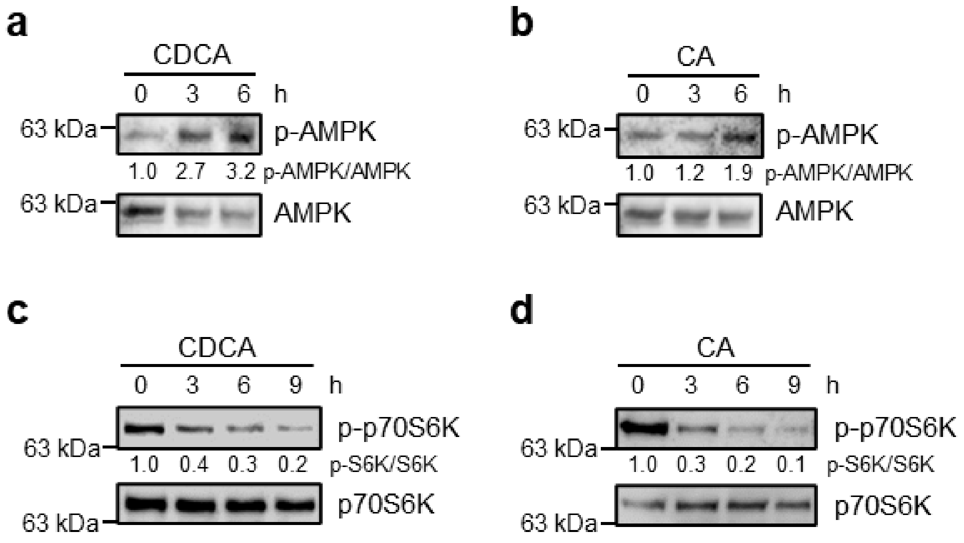

Supplementary Fig. 9. (a-d) HepG2 cells were treated with 200 μM CDCA or 750 μM CA for the indicated times. Cell lysates were immunoblotted with anti-p-AMPK, anti-AMPK, anti-p-p70S6K, and anti-p70S6K antibodies. Numbers below immunoblot bands indicate the fold changes normalized to control band intensities. Results are representative of at least three independent experiments.
